# Leveraging National Cancer Institute Programmatic Collaboration for Uterine Cervix Cancer Patient Accrual in Puerto Rico

**DOI:** 10.3389/fonc.2018.00102

**Published:** 2018-04-10

**Authors:** Charles A. Kunos, Holly A. Massett, Annette Galassi, Joan L. Walker, Marge J. Good, Luis Báez Díaz, Worta McCaskill-Stevens

**Affiliations:** ^1^Division of Cancer Treatment and Diagnosis, National Cancer Institute, Bethesda, MD, United States; ^2^Center for Global Health, National Cancer Institute, Bethesda, MD, United States; ^3^Gynecologic Oncology Section, Stephenson Cancer Center, University of Oklahoma, Oklahoma City, OK, United States; ^4^Division of Cancer Prevention, National Cancer Institute, Bethesda, MD, United States; ^5^Minority/Underserved NCI Community Oncology Research Program, San Juan, PR, United States

**Keywords:** Puerto Rico, uterine cervix cancer, cervical cancer, accrual, barriers to participation, radiotherapy

## Abstract

Women in the U.S. Commonwealth of Puerto Rico (PR) have a higher age-adjusted incidence rate for uterine cervix cancer than the U.S. mainland as well as substantial access and economic barriers to cancer care. The National Cancer Institute (NCI) funds a Minority/Underserved NCI Community Oncology Research Program in PR (PRNCORP) as part of a national network of community-based health-care systems to conduct multisite cancer clinical trials in diverse populations. Participation by the PRNCORP in NCI’s uterine cervix cancer clinical trials, however, has remained limited. This study reports on the findings of an NCI site visit in PR to assess barriers impeding site activation and accrual to its sponsored gynecologic cancer clinical trials. Qualitative, semi-structured individual, and group interviews were conducted at six PRNCORP-affiliated locations to ascertain: long-term trial accrual objectives; key stakeholders in PR that address uterine cervix cancer care; key challenges or barriers to activating and to enrolling patients in NCI uterine cervix cancer treatment trials; and resources, policies, or procedures in place or needed on the island to support NCI-sponsored clinical trials. An NCI-sponsored uterine cervix cancer radiation–chemotherapy intervention clinical trial (NCT02466971), already activated on the island, served as a test case to identify relevant patient accrual and site barriers. The site visit identified five key barriers to accrual: (1) lack of central personnel to coordinate referrals for treatment plans, medical tests, and medical imaging across the island’s clinical trial access points; (2) patient insurance coverage; (3) lack of a coordinated brachytherapy schedule at San Juan-centric service providers; (4) limited credentialed radiotherapy machines island-wide; and (5) too few radiology medical physicists tasked to credential trial-specified positron emission tomography scanners island-wide. PR offers a unique opportunity to study overarching and tactical strategies for improving accrual to NCI-sponsored gynecologic cancer clinical trials. Interview findings support adding and re-tasking personnel for coordinated trial-eligible patient referral, accrual, and treatment.

## Introduction

Cancer incidence and mortality are disproportionately higher among racial, ethnic, and underserved groups in the U.S. (1), often due to a lack of health-care access and low socioeconomic status (e.g., income and education) (2). According to the U.S. Census Bureau (3–6), residents of the U.S. Commonwealth of Puerto Rico (PR) are Hispanic/Latino (99%), speak a language other than English in their home (95%), have a median household income of $19,350 which means 46% of them live in poverty (compared with 10% of non-Hispanic Whites on the U.S. mainland), and only about a quarter (24%) of the population earns a Bachelor’s degree or above (compared with 36% of non-Hispanic Whites on the mainland). PR’s population of 1.9 million women (7) has an age-adjusted incidence rate for uterine cervix cancer that exceeds the U.S. mainland’s rate [12.2 per 100,000 people versus 7.5, respectively (8)]. In PR’s largest municipality, San Juan, the number of new uterine cervix cancer diagnoses is on the rise, increasing 10% (from 31 to 34 cases) between 2010 and 2012 (9). Two other municipalities also saw gains in uterine cervix cancer cases of at least 10% during this same time frame (9).

Cancer clinical trials provide the necessary research to identify better ways to prevent, treat, control, and cure cancer. It is critical that populations disproportionately affected by cancer are included in trials to ensure that new discoveries are relevant to these groups (10, 11). To address disparities in such research, the National Cancer Institute (NCI) funds the Minority/Underserved NCI Community Oncology Research Program (MU-NCORP), a national network of community-based health-care systems that conduct multisite cancer clinical trials in diverse populations (12). PR is one of 12 MU-NCORPs and is called the Puerto Rico MU-NCORP (PRNCORP).

**Figure 1:**
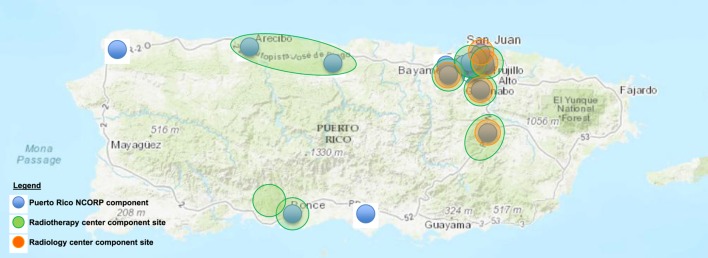
Puerto Rico NCI Community Oncology Research Program (NCORP) components in 2017.

The PRNCORP has a large footprint on the island that includes 17 cancer treatment sites (Figure 1). It is PR’s primary cancer clinical trial organization. Despite the high burden of uterine cervix cancer in PR, there has been almost no participation by the PRNCORP in NCI’s uterine cervix cancer clinical trials. Between 2006 and 2016, NCI activated eight uterine cervix cancer clinical trials. Only one of these trials was activated in the PRNCORP, and no women on the island enrolled onto this trial (13). Reasons for the negligible participation in these eight cancer clinical trials are unknown, but there is substantial general evidence in the literature that organizational barriers (e.g., capacity and costs) prohibit sites from opening available trials that might benefit their patients (14); and patients do not enroll due to limited health-care access, low awareness or fear of trials, and lack of physician referral, further exacerbated by low-socioeconomic status and poverty (15, 16). Underrepresentation of Hispanic/Latino women on NCI uterine cervix clinical trials remains a persistent problem—whereas the uterine corpus cancer and uterine cervix cancer incidences were high for Hispanics in 2010 (13.0 and 21.6%, respectively), the observed accrual of Hispanic/Latino women to uterine corpus or uterine cervix trials between 2003 and 2012 was low (5.1 and 14.3%, respectively) (17).

Given the low participation of the PRNCORP in uterine cervix cancer clinical trials and this cancer’s high incidence but low accrual among Hispanic/Latino women on the island, the NCI undertook a multimethod programmatic research effort to identify barriers to clinical trial site activation and patient enrollment in NCI-sponsored gynecologic cancer trials in PR. This study summarizes the findings from the research and presents recommendations and actions taken to address the identified barriers. This research effort was conducted in March 2017 and was a collaborative venture between NCI’s Cancer Therapy Evaluation Program (CTEP), the Division of Cancer Prevention (DCP, which oversees NCI Community Oncology Research Program in PR), and the principal investigator (PI) and research team at the PRNCORP.

## Materials and Methods

A five-member, joint CTEP, and DCP team conducted an onsite visit in March 2017 at six PRNCORP-affiliated locations on the island: (1) Centro Compresivo de Cáncer de le Universidad de Puerto Rico (CCCUPR) in San Juan, (2) CCCUPR Radiotherapy Center in San Juan, (3) Precise Radiation Oncology in Bayamón, (4) PET Imaging Radiology PSC in Bayamón, (5) San Patricio MEDFLIX in Guaynabo, and (6) HIMA San Pablo in Caguas. The site visits involved a multimethod qualitative research approach including: (1) 6 structured one-on-one interviews with health-care providers; (2) 2 small group discussions with 9 clinical research associates (CRAs) and 10 oncologists; and (3) tours of the CCCUPR and HIMA San Pablo Hospital facilities, 3 PRNCORP-affiliated radiotherapy centers, and 4 positron emission tomography (PET) centers on the island. In aggregate, the interviews and group discussions aimed to answer four key research questions: Who are the key stakeholders in PR that address uterine cervix cancer patient care? How can NCI maximize its relationship with them to enhance patient awareness of NCI clinical trials? What are the key challenges or barriers to activating NCI uterine cervix cancer treatment trials at PR sites and to enroll eligible patients? What resources, policies, or procedures are in place or are in need on the island to support NCI-sponsored clinical trials?

To explore the barriers challenging the PRNCORP with respect to activating and accruing patients to NCI uterine cervix cancer clinical trials, the research team focused on one NCI-sponsored phase II therapeutic cancer clinical trial as its test case. The NRG Oncology GY006 clinical trial (NCT02595879) was selected, as it is a randomized phase II trial evaluating the experimental therapy of triapine–cisplatin–radiotherapy versus standard-of-care cisplatin–radiotherapy in women with advanced-stage uterine cervix cancer. This trial was a good choice as it was active for accrual in the PRNCORP at the time of the site visit and includes the typical complexities of a uterine cervix cancer trial, such as coordinated 8-week radiotherapy, brachytherapy, and chemotherapy outpatient therapy visits.

### Analysis Plan

Using interview and visit notes, researchers mapped by hand the overall flow of patients, providers, and services that make up the communications and medical care processes among the practitioners, facilities, and patients involved. Summary findings were then reviewed with five PR gynecologic oncologists and PRNCORP stakeholders during an exit interview (conducted before concluding the site visit) to ensure face validity of the findings and to allow discussion of possible solutions to known challenges, including potential infrastructure changes based on observed findings.

## Results

Overall, the site visit identified five key high-level barriers to accrual: (1) lack of central personnel to coordinate referrals for treatment plans, medical tests, and medical imaging across the island’s clinical trial access points; (2) patient insurance coverage; (3) lack of a coordinated brachytherapy schedule at San Juan-centric service providers; (4) limited credentialed radiotherapy machines island-wide; and (5) too few radiology medical physicists tasked to credential trial-specified PET scanners island-wide. Each are described below in detail.

### Difficulties With Coordination and Clinical Trial Access Points

#### Description of Long-Term Accrual Objectives

PRNCORP respondents reported a continued commitment to the NCI’s overall clinical trial enterprise, matching the mission for NCORP scientific programs of cancer prevention and symptom intervention trials and of therapeutic cancer clinical trials to provide a unique research portfolio island-wide.

#### Accrual Achievements

Between 2014 and 2017, a single person was accrued in PR to a randomized phase II clinical trial for advanced-stage uterine cervix cancer.

#### Sites and Community Practice

Currently, the PRNCORP serves Puerto Rican women at 17 cancer treatment sites dispersed across the island (Figure 1). For these 17 clinical sites, there is one gynecologic medical oncologist who serves as the PRNCORP PI. The PI directly supervises 5 of the 17 sites and coordinates most (>80%) patient referrals for gynecologic cancer treatment in clinical trials across the island’s 17 PRNCORP sites, with the assistance of two research nurses and nine CRAs. For the remaining 12 sites, there are 5 gynecologic oncologists or other medical oncologists who are also active investigators in the PRNCORP and manage patients who are potentially eligible for clinical trials with the PRNCORP’s CRAs. Among the PI and the five gynecologic oncologists, care is provided for the majority (>90%) of uterine cervix cancer patients on the island.

#### Navigation and Access to Standard-of-Care

Standard-of-care treatment for women with advanced-stage uterine cervix cancer typically involves (a) office visits or examinations under anesthesia for clinical assessment, biopsy, and treatment planning; (b) medical tests for renal and hepatic function as well as pregnancy status; (c) risk assessments by gynecologic, medical, and radiation oncologists; and (d) ancillary medical imaging studies such as contrast-enhanced computed tomography (CT) or whole-body PET-CT scans. According to respondents, the diagnosis and clinical staging of uterine cervix cancer in PR relies upon input from multiple health-care providers. To receive treatment, patients often must travel across the island to accommodate non-overlapping health-care provider clinical schedules, resulting in substantial delays in cancer care planning and catastrophic-need designation (a PR Medicaid category that activates specialty medical care benefits on the island). Because of cancer care navigational delays, medical tests or imaging required for trial eligibility often occur outside the window of time in which the trial requires them—termed “out-of-window” in trial shorthand. Respondents identified out-of-window medical tests or imaging intervals as the primary obstacle to patient accrual to uterine cervix clinical trials. It is believed that these circumstances arise from the impractical logistics of women having to self-navigate their cancer care. In PR, there are no registered nurse navigators trained in women’s cancer to guide patients through the complex roadmap for expert medical care. Other issues such as patient insurance coverage [n.b., 60% of women in PR have Medicaid coverage (18)], low cancer care service reimbursement, cancer care delivery culture resulting in a low rate of internal referrals, and a lack of an island-wide electronic medical record were also reported as adding to the complexity of delivering gynecologic cancer care in PR.

#### Clinical Trial Referral Coordination

**Figure 2:**
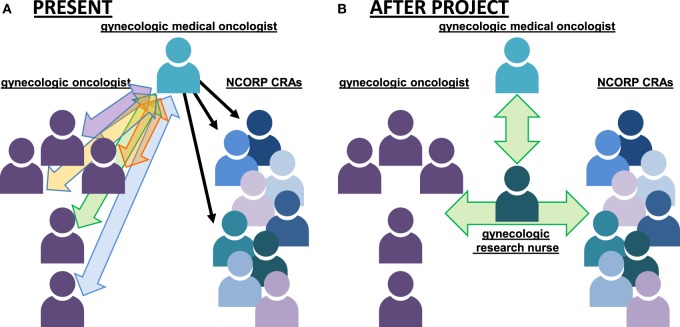
Diagram of referral coordination for women with gynecologic cancers in Puerto Rico (PR) in 2017. **(A)** Individual gynecologic cancer patient referrals pass through the PR Minority Underserved NCORP gynecologic medical oncologist to the CRAs in 2017. **(B)** Under a pilot study, a full-time effort gynecologic research nurse will provide a “go-to” trial champion and coordinate cancer care among the indicated providers. Abbreviations: NCORP, NCI Community Oncology Research Program; CRA, clinical research associate.

Figure 2A illustrates the overall approach to gynecologic cancer care and coordination in PR. As shown in the figure, women with advanced-stage uterine cervix cancer in PR typically proceed stepwise from a gynecologic oncologist for consultation and any diagnostic or surgical procedures to the PRNCORP gynecologic medical oncologist for consultation and chemotherapy plans. The PRNCORP CRAs then implement the cancer care plans for the patients. Patients then return to their gynecologic oncologists for long-term follow-up. With respect to clinical trial recruitment, almost all respondents interviewed agreed that this multi-health-care provider approach makes it difficult to coordinate the patient referral process for clinical trial participation before the start of patients’ active treatment. Furthermore, respondents indicated that the PRNCORP lacks a single point-of-contact individual who is responsible for coordination of clinical trial enrollment when an eligible gynecologic cancer patient is identified. As Figure 2A shows, the gynecologic medical oncologist sits atop the PRNCORP referral hierarchy and the nine CRAs report exclusively to this person and do not directly field referrals from the five gynecologic oncologists on the island. Instead, referrals from these five gynecologic oncologists are made directly to the PRNCORP gynecologic medical oncologist who is then the sole consultant for a patient’s initial cancer care plan and presentation of therapeutic clinical trials. In effect, a bottleneck builds up whereby all referred patients’ care is coordinated *via* the one gynecologic medical oncologist who then must directly oversee the nine CRAs implementing care island-wide.

### Insurance Coverage Concerns

#### Medical Care

Respondents reported several reasons for slow accrual across the island. The most commonly cited reasons were systemic in nature—having to do with administrative, access, and quality control issues. The top reasons noted were (a) delays due to administrative problems at the site level for a patient’s letter of medical catastrophe and (b) protracted eligibility reviews by community physicians that result in patients missing the window of opportunity for trial-specific medical tests. Sixty percent of women on the island access health-care through Mi Salud, the PR Medicaid acute, primary, and specialty medical care delivery system (18). Under Mi Salud health-care provider contracts, cancer patients must first have catastrophic-need designation to activate their specialty medical care benefits. Moreover, the catastrophic-need designation must be renewed every 6 months to maintain cancer care. This unique catastrophic-need designation qualifier distinguishes PR from other U.S. mainland clinical research sites. Safety or toxicity issues were not perceived as barriers to accrual, which differs from findings of prior NCI evaluations for slow accrual (19).

### Lack of Coordinated Brachytherapy Schedules

On the island, there are up to 10 radiation oncologists that report gynecologic brachytherapy experience for the treatment of uterine cervix cancer. Outpatient brachytherapy delivery for this disease appears haphazard on the island, variable by practitioner, and again is San Juan-centric {three centers [3 (18%) of 17 PRNCORP sites]}, making it difficult for the PRNCORP to schedule their patients to brachytherapy in a coordinated manner. It is said that up to 70% of women with uterine cervix cancer may not receive standard-of-care brachytherapy due to their lack of awareness of the vital importance of this treatment. It is also reported that the need to travel from the western and southern island sites for access impedes brachytherapy delivery.

### Limited Credentialed Radiotherapy Machines

Respondents from the PRNCORP consistently noted the lack of access to credentialed, quality assurance-monitored radiotherapy centers for their patients. Of the 17 clinical sites, only eight (53%) offer local external beam radiotherapy centers to serve their gynecologic cancer patients. Only three (38%) of these radiotherapy centers are monitored by the Imaging and Radiation Oncology Core (IROC)-Houston Radiation Physics Center for quality assurance of radiation machine output and dose delivery, a requirement of all NCI-sponsored clinical trials. These radiation machines are San Juan-centric, which makes them difficult to access from other points on the island. Other trial-appropriate radiation machines exist on the island and are in uterine cervix cancer patient “hot-spots,” but these are not appropriately credentialed for NCI-sponsored clinical trials. Respondents indicated that health-care provider champions are in place in these island hot-spots and are motivated to present uterine cervix cancer trials to eligible patients should credentialed radiation machines become available in their area.

### Too Few Radiology Medical Physicists

Respondents noted a limited number of credentialed diagnostic radiology PET centers. The island has five PET centers clustered in the greater San Juan city region; only one has passed trial-specific quality standards set forth by the IROC-Philadelphia imaging center which oversees PET scanner quality control. The one “passing” center in PR does not accept Medicaid, the predominant form of medical insurance for over 60% of women on the island. Medicaid-accepting PET centers do exist, but they encounter the pitfall of not have sufficient funds to hire off-site medical physicists to perform trial-specific credentialing tasks. While IROC-Philadelphia provides a credentialing checklist and learning opportunities, PET center staff in PR may not have sufficient practical experience in NCI-sponsored trial-specific credentialing tasks.

## Discussion

This article describes the findings from a site visit by NCI representatives to the MU-NCORP on the island of PR in an effort to explore barriers to patient accrual to NCI-sponsored uterine cervix cancer treatment trials. Overwhelmingly, the site visit confirmed the commitment of the PRNCORP team to support clinical research and to access trials for women with uterine cervix cancer. The site visit also found that there are five key systemic barriers that impede the PRNCORP team from referring eligible patients to NCI-sponsored uterine cervix cancer clinical trials: (1) lack of central personnel to facilitate insurance requirements and coordinate patient referral to gynecologic cancer trials across the island in a timely manner such that eligible patients are aware of trials, receive personal assistance in navigating the process, and enroll within an eligible timeframe; (2) insurance coverage as an accrual barrier; (3) lack of an island-wide gynecologic brachytherapy service to support their patients’ needs; (4) lack of credentialed radiotherapy machines island-wide; and (5) lack of credentialed PET scanners island-wide. All barriers contribute meaningfully to the finding that all women in PR may not have timely access to standard-of-care treatment usually readily available on the U.S. mainland.

Collectively, NCI representatives and PRNCORP team members worked together in exit interviews to develop a set of feasible recommendations to address each of the four barriers identified. First, the teams believed that the PRNCORP needs to hire a point person to help manage patient referrals from health-care providers around the island as well as to help eligible patients navigate the catastrophic-need designation and clinical trial treatment processes. Specifically, this person would (1) function as the PRNCORP affiliated, go-to contact for gynecologic oncologists, radiation oncologists, and CRAs on the island (with a single point of contact) for the referral of potentially trial-eligible patients; (2) offer patient navigation to support women’s decision-making processes for their treatment and understanding of clinical trial options; and (3) ensure that the trial-related needs of patients and health-care providers are addressed in a timely fashion—possessing cancer care-appropriate skill and aiding quick adverse event management. The PRNCORP envisions a dedicated gynecologic research nurse as being best positioned to manage both tasks. This individual would report directly to the PRNCORP team to reduce the apparent administrative bottleneck and time constraints on the PI (see Figure 2B). The second recommendation, addressing the haphazard accessibility of brachytherapy on the island, suggests that the PRNCORP negotiate with brachytherapy-capable radiotherapy center staff to hold a set of brachytherapy slots on a specific day of the week in the San Juan area to accommodate multiple patients who travel from a distance, with a particular emphasis to meet the requirements of NCI-sponsored uterine cervix cancer clinical trials. The third recommendation, addressing accessibility of credentialed radiotherapy machines and diagnostic radiology PET centers, suggests that the PRNCORP identify already-affiliated medical physicists that are tasked with credentialing PRNCORP-affiliated radiotherapy and imaging centers, again with a particular importance to meet the standards of NCI-sponsored clinical trials.

The final two recommendations address the importance of good radiotherapy machine or radiology instrument quality control programs within trials (20). Trial quality control might not stipulate which machine or how often an instrument should be credentialed, but rather affirm that local quality control programs match national standards. In the context of clinical trials, quality control programs ensure that aspects of the diagnostic or treatment delivery chain minimally affect the capacity to answer the clinical or scientific trial objective. Thus, the NCI and PRNCORP teams recommend: (a) re-tasking an existing PRNCORP-affiliated radiation medical physicist to perform phantom irradiation and to collect and to submit quality assurance data at all eight radiotherapy centers in PR for IROC-Houston (approximately 10 weeks of effort) and (b) funding a PRNCORP-affiliated part-effort imaging medical physicist to perform phantom scanning and to submit image quality data at all five PET centers for IROC-Philadelphia (approximately 5 weeks of effort). Within a reasonable budget, re-tasked medical physicists (already residing in PR) would be able to credential existing machines and instruments without too great a burden on centers such that it would not disrupt normal business but set tolerance limits appropriate for the accuracy required within an NCI-sponsored trial. NCI-sponsored radiotherapy trials require participating centers to audit regularly their machine output and dosimetry to demonstrate a prespecified level of radiation dose accuracy. In some trial instances, like in the NRG Oncology GY006 clinical trial, non-adherence to quality control parameters (e.g., radiotherapy or PET instrument limits) might be more impactful on the determination of treatment response than the impact of experimental trial combinations simply due to an out-of-range tool obscuring trial findings.

This research was qualitative in nature and therefore not generalizable to other NCORP sites or disease types. The findings and recommendations are specific to the systemic barriers identified at those locations visited across PR and very well could have missed additional concerns outside of the scope of this research intent. In fact, the literature clearly identifies a whole host of additional barriers that can impact both site activation and patient accrual. However, the value of qualitative research is its ability to explore topics in depth and to discuss in detail potential solutions that are tailored to findings.

Overall, this research highlights the importance of conducting onsite visits to regions where the incidence of cancer is high, and despite the interest and potential availability of clinical trials, eligible patients are not enrolled. Within a manageable timeframe of 1 week, the joint efforts of the NCI and PRNCORP teams resulted in a well-defined list of accrual barriers and a respective list of recommendations to address each. Moreover, the NCI has since committed resources to fund each recommendation as part of a 2-year demonstration project to assess feasibility and impact of accrual for each of the four recommendations.^1^ All efforts to reduce the barriers to uterine cervix cancer treatment trials will be documented and evaluated for their successes and lessons learned, and will be reported out to ensure transparent communication around their impact on patient accrual.

## Ethics Statement

This original research was carried out in accordance with the guidelines for clinical observational research of the National Cancer Institute. No human subject research was conducted.

## Author Contributions

Conception and design: CK, HM, LD, and WM-S. Financial support: CK and WM-S. Administrative support: HM, AG, MG, and JW. Provision of study materials or patients: CK, HM, and WM-S. Collection and assembly of data: CK, AG, MG, JW, and WM-S. Data analysis and interpretation; manuscript writing; and final approval of manuscript: CK, HM, AG, MG, JW, LD, and WM-S.

## Conflict of Interest Statement

The authors declare that the research was conducted in the absence of any commercial or financial relationships that could be construed as a potential conflict of interest.

## Footnotes

^1^These recommendations were established prior to Hurricane Maria hitting PR in September 2017 and the resulting impact of the natural disaster. NCI recognizes the uncertainty of implementing the demonstration project throughout the island in a timely manner to achieve the intended goals and will work with the PRNCORP adjust accordingly.

## References

[1] American Cancer Society. Cancer Facts and Figures for Hispanics and Latinos. American Cancer Society Repository (2016). Available from: https://www.cancer.org/research/cancer-facts-statistics/hispanics-latinos-facts-figures.html (Accessed: November 02, 2017).

[2] National Cancer Institute. Cancer Health Disparities. National Cancer Institute Repository (2017). Available from: https://www.cancer.gov/about-nci/organization/crchd/cancer-health-disparities-fact-sheet (Accessed: November 02, 2017).

[3] United States Census Bureau. Income and Poverty in the United States: 2014. United States Census Bureau Repository (2015). Available from: https://www.census.gov/content/dam/Census/library/publications/2015/demo/p60-252.pdf (Accessed: November 02, 2017).

[4] United States Census Bureau. Income and Poverty in the United States: 2015. United States Census Bureau Repository (2016). Available from: https://www.census.gov/library/publications/2016/demo/p60-256.html (Table A1) (Accessed: November 02, 2017).

[5] United States Census Bureau. Educational Attainment in the United States: 2015. United States Census Bureau Repository (2016). Available from: https://www.census.gov/content/dam/Census/library/publications/2016/demo/p20-578.pdf (Accessed: November 02, 2017).

[6] United States Census Bureau. QuickFacts: Puerto Rico. United States Census Bureau Repository (2017). Available from: https://www.census.gov/quickfacts/PR (Accessed: November 02, 2017).

[7] United States Census Bureau. 2010 Census of Population and Housing, Summary Population and Housing Characteristics: CPH-1-53 Puerto Rico. United States Census Bureau Repository (2012). Available from: https://www.census.gov/prod/cen2010/cph-1-53.pdf (Accessed: November 02, 2017).

[8] Pan American Health Organization. World Health Organization Regional Office for the Americas: Puerto Rico CANCER Profile 2013. Pan American Health Organization Repository (2013). Available from: http://www.paho.org (Accessed: November 02, 2017).

[9] Registro Central De Cáncer De Puerto Rico. Tasas y Mapas – Registro de Cáncer de Puerto Rico. Registro Central De Cáncer De Puerto Rico Repository. (2017). Available from: http://www.rcpr.org/Datos-de-Cáncer/Tasas-y-Mapas (Accessed: November 02, 2017).

[10] National Cancer Institute. Cancer Therapy Evaluation Program. National Cancer Institute Repository (2017). Available from: http://ctep.cancer.gov (Accessed: November 02, 2017).

[11] Duma N Aguilera JV Paludo J Haddox CL Velez MG Wang Y Representation of minorities and women in oncology clinical trials: review of the past 14 years. J Oncol Pract (2018) 14(1):e1–10. 10.1200/JOP.2017.025288 29099678

[12] National Cancer Institute. National Cancer Institute Community Oncology Research Program (NCORP). National Cancer Institute Repository (2017). Available from: http://ncorp.cancer.gov (Accessed: 02 November, 2017).

[13] Kunos CA Personal Communication: Protocol #9434. Bethesda, MD: National Cancer Institute (2017). Available from: (Accessed: November 02, 2017).

[14] Somkin CP Ackerson L Husson G Gomez V Kolevska T Goldstein D Effect of medical oncologists’ attitudes on accrual to clinical trials in a community setting. J Oncol Pract (2013) 9(6):e275–83. 10.1200/JOP.2013.001120 24151327 PMC5706122

[15] Mills EJ Seely D Rachlis B Griffith L Wu P Wilson K Barriers to participation in clinical trials of cancer: a meta analysis and systematic review of patient-reported factors. Lancet Oncol (2006) 7(2):141–8. 10.1016/S1470-2045(06)70576-9 16455478

[16] Unger JM Gralow JR Albain KS Ramsey SD Hershman DL Patient income level and cancer clinical trial participation: a prospective survey study. JAMA Oncol (2016) 2(1):137–9. 10.1001/jamaoncol.2015.3924 26468994 PMC4824189

[17] Mishkin G Minasian LM Kohn EC Noone AM Temkin SM. The generalizability of NCI-sponsored clinical trials accrual among women with gynecologic malignancies. Gynecol Oncol (2016) 143(3):611–6. 10.1016/j.ygyno.2016.09.026 27697287

[18] Portela M Sommers BD. On the outskirts of national health reform: a comparative assessment of health insurance and access to care in Puerto Rico and the United States. Milbank Q (2015) 93:584–608. 10.1111/1468-0009.12138 26350931 PMC4567854

[19] Massett HA Mishkin G Rubinstein L Ivy SP Denicoff A Godwin E Challenges facing early phase trials sponsored by the National Cancer Institute: an analysis of corrective action plans to improve accrual. Clin Cancer Res (2016) 22(22):5408–16. 10.1158/1078-0432.CCR-16-0338 27401246 PMC5226927

[20] Ibbott GS Haworth A Followill DS. Quality assurance for clinical trials. Front Oncol (2013) 3:311. 10.3389/fonc.2013.00311 24392352 PMC3867736

